# No Tillage With Plastic Re-mulching Maintains High Maize Productivity *via* Regulating Hydrothermal Effects in an Arid Region

**DOI:** 10.3389/fpls.2021.649684

**Published:** 2021-04-09

**Authors:** Wen Yin, Qiang Chai, Yao Guo, Hong Fan, Zhilong Fan, Falong Hu, Cai Zhao, Aizhong Yu, Jeffrey A. Coulter

**Affiliations:** ^1^Gansu Provincial Key Laboratory of Aridland Crop Science, Lanzhou, China; ^2^College of Agronomy, Gansu Agricultural University, Lanzhou, China; ^3^Department of Biological Systems Engineering, Washington State University, Pullman, WA, United States

**Keywords:** maize, plastic mulch, soil moisture, soil temperature, crop production, economic benefit

## Abstract

Plastic is a valuable mulching measure for increasing crop productivity in arid environments; however, little is known about the main mechanism by which this valuable technology actuates spatial–temporal changes in soil hydrothermal effect. So a 3-year field experiment was conducted to optimize soil hydrothermal effect of maize field with three plastic mulched management treatments: (1) no tillage with plastic re-mulching (NM), (2) reduced tillage with plastic mulching (RM), and (3) conventional tillage with annual new plastic mulching (CM). The results showed that NM treatment increased soil water content by 6.6–8.4% from maize sowing to seedling stage, than did CM, and it created a good soil moisture environment for sowing of maize. Also, NM had greater soil water content by 4.8–5.6% from maize silking to early-filling stage than had CM, and it made up for the abundant demand of soil moisture for the vigorous growth of maize filling stage. The NM treatment increased water consumption (WC) before maize big-flare stage, decreased WC from big-flare to early-filling stage, and increased WC after early-filling stage. So NM treatment effectively coordinated water demand contradiction of maize at entire growing season. NM decreased soil accumulated temperature (SAT) by 7.0–13.0% at maize sowing to early-filling stage than did CM, but NM had little influence on the SAT during filling stage. In particular, the treatment on NM had smaller absolute values of air–soil temperature differences than RM and CM treatments during maize filling stage, indicating that NM treatment maintains the relative stability of soil temperature for ensuring grain filling of maize. The NM treatment allowed the maize to grow in a suitable hydrothermal status and still maintained high yield. In addition, NM treatment obtained higher net income and rate of return by 6.4–11.0% and 44.1–54.5%, respectively, than did CM, because NM treatment mainly decreased the input costs for plastic and machine operations. Therefore, the NM treatment can be recommended as a promising technique to overcome simultaneous heat stress and water shortage in arid environments.

## Introduction

The growing human population needs more resources to produce food (Godfray et al., 2010), and in highly populated semiarid or arid regions, water shortage is a challenge affecting agricultural development (Tilmana et al., 2011). Arid and semiarid areas cover more than two fifths of the world’s arable land and feed more than one third of the world’s population (Farooq and Siddique, 2016). So agricultural production in these regions plays a major role in the global food supply. In typical arid regions such as the oasis irrigation of northwestern in China, water shortages and extreme air temperatures have a huge impact on agricultural production (Chai et al., 2014; Yin et al., 2016). Low air temperature in the spring, which always drops suddenly at night, has an adverse effect on seed germination, root activity, crop establishment, and growth (Chen et al., 2011). As a result, these adverse effects can weaken the use of soil water by crops, thus further exacerbating drought stress (Song et al., 2013). Optimized hydrothermal management practices are very important for crop cultivation in arid regions because they can enhance crop productivity (Zhao et al., 2014; Yin et al., 2016), which can guarantee grain supply and strengthen sustainable agricultural development (Lobell et al., 2008; Gan et al., 2013).

A fully mulched system with plastic, a promising water harvesting and conservation practice, is a strategy to capture rainwater and store irrigation water (Li et al., 2013; Zhang et al., 2019). It has become a common measure for crop production and has been widely applied in arid and semiarid environments (Gan et al., 2013; Yin et al., 2018b). This practice can enhance the effective utilization of soil water by inhibiting soil evaporation, strengthening water conservation and harvesting, and decreasing the wastage of heat loss from the soil to the atmosphere (Sarkar et al., 2007; Zhou et al., 2009). At present, the plastic mulched system has been widely used in the oasis irrigation regions of northwestern China because of the enhanced efficiency in water harvesting and improvements in grain yield and resource use efficiency of maize (Chai et al., 2014; Yin et al., 2020b). However, in recent years, the precocity of maize with plastic mulch is remarkably common, and some researchers believed that this phenomenon is premature aging rather than early maturation, and it is a precursor to reduced yield (Bu et al., 2013; Yin et al., 2020b). The reason for this precocity is that transparent plastic mulching often causes extreme high soil temperature in the root zone of maize at the reproductive growth period, leading to senescence of roots and leaves for maize and weakening the stability of yield performance (Bu et al., 2013). In addition, the large amount of plastic for maize cultivation in arid oasis irrigated areas has adverse environmental impacts, such as greenhouse gas emissions, white pollution, and soil degradation, which are also a major factor affecting yield stability (Liu et al., 2014). Thereby, it is important to develop an efficient plastic mulched management practice to maintain maize yield and use of water resource while reducing the input of plastic.

Tillage practice can significantly affect water use and yield performance of crops *via* regulating hydrothermal effect of soil (Licht and Al-Kaisi, 2005; Sarkar and Singh, 2007). No or reduced tillage can increase the available moisture content across the soil profile (Lampurlanés et al., 2016); optimize soil physical (Czyz and Dexter, 2009), chemical (Sabine et al., 2013), and biological properties (Stenberg et al., 2000); and regulate soil heat (Sarkar and Singh, 2007), thus enhancing crop production (Fernández-García et al., 2013), especially in no or reduced tillage with straw retention. No tillage is considered as an effective strategy to enhance soil water-holding capacity, reduce soil evaporation, and optimize soil heat status (Lampurlanés et al., 2016; Dai et al., 2021), thus improving the sustainability of crop production. However, the effect of no tillage was integrated with plastic mulching system on the growth; and development of maize is rarely reported in oasis irrigation areas of northwestern China where maize cultivation must rely on plastic mulching. Therefore, it is important to develop a system on no tillage with plastic re-mulching to maintain maize productivity through the optimization of soil hydrothermal properties. We propose a promising and valuable system in which plastic secondary recycling is used for maize production, which is based on existing findings on the regulatory mechanisms for soil hydrothermal effect (Yin et al., 2016, 2020b). The central hypothesis of this promising and valuable system can decrease the change of air–soil temperature amplitude and coordinate water demand contradiction to maintain high maize productivity while reducing the input of plastic in arid regions.

Some studies on crop production were aimed at optimizing the soil hydrothermal characteristics *via* mulching mainly focusing on straw (Sekhon et al., 2008; Chen et al., 2010). Few researches have explored the integrated effects of no tillage with plastic mulched management on soil hydrothermal characteristics in a crop production system. An innovative system on no tillage with plastic re-mulching in maize field may have the potential to create an excellent soil microenvironment to keep more vigorous growth and development of maize *via* optimizing soil moisture and heat status. The mechanism by which this innovative system drives spatial–temporal changes in soil temperature and moisture that affect maize cultivation is not well explained. Learning more about this mechanism will provide a theoretical and practical basis for improving plastic mulched management to boost maize productivity. So in this study, we aimed to (1) investigate the effects of plastic mulched management practices on soil moisture and temperature in maize farmland; (2) evaluate the effects of plastic mulched management practices on yield performance of maize; and (3) verify the feasibility of no tillage with plastic re-mulching in maize production at semiarid and arid regions or others similar climatic regions.

## Materials and Methods

### Description of Experimental Sites

**FIGURE 1:**
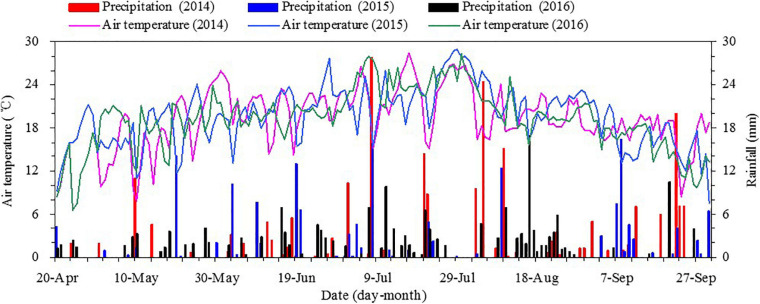
Daily mean air temperature and total precipitation during the growing season from 2014 to 2016 at the Wuwei Experimental Station in northwestern China.

A field experiment was conducted at the Wuwei Oasis Agricultural Comprehensive Experimental Station, Gansu Agricultural University of Northwestern China, in 2013–2016; and a preparatory experiment was conducted in 2013, to form the previous residual plastic re-mulching in the field for the implementation of the experimental treatments in 2014, 2015, and 2016. The soil at the experimental area is classified as an Aridisol, and the area is a cold and temperate arid climate zone. Over the last 50 years of this experimental area, mean annual sunshine duration was > 2,800 h, the mean annual accumulated temperature (>10°C) was > 2,800°C, the annual total solar radiation was also > 5,500 MJ m^–2^, and the mean annual frost-free season was > 150 days. So the sunshine and heat conditions in this experimental area were suitable for maize cultivation. In 2014, 2015, and 2016, the rainfall of growing season for maize was 242, 160, and 182 mm, respectively, and the distribution of rainfall and air temperature is shown in Figure 1.

### Field Experimental Design and Management

The 3-year field experiment was conducted with a randomized complete block design and with three replicates. A pre-experiment was carried out in 2013 to form previous residual plastic re-mulching (i.e., plastic mulching was reused for the second year) as preparation for the implementation of the experimental treatments in 2014–2016. The three treatments were as follows: (1) no tillage with plastic re-mulching, soil was mulched with new plastic in the previous year, and the plastic was preserved with no tillage and sowing directly on the residual plastic film in the following spring (no tillage with plastic mulching, NM); (2) no tillage with plastic mulching after maize was harvested in autumn, and new plastic was mulched in next spring after the old plastic was removed off the field and rotary ploughed (reduced tillage with plastic mulching, RM); and (3) conventional tillage (the depth is 30 cm) after maize was harvested and the old plastic was removed off the field in autumn, and new plastic was mulched in next spring before maize sowing (conventional tillage with plastic mulching, CM, the control). The experimental design of NM and RM treatments was alternated over 2 years. The description of the experimental design is shown in Table 1. For the NM treatment, fertilizers were applied and maize was planted on the previous residual plastic by a roller dibbler. For RM and CM treatments, the experimental soil was covered by new plastic after soil was fertilized, rotary tillage, and compacted and maize was planted, consistent with NM treatment.

**TABLE 1:** Yearly calendar diagram, which sets out the annual cycle of tillage and plastic practices for maize cultivation, at Wuwei Experimental Station, northwestern China, 2013–2016.

Year	Growing stage	Tillage and mulching practices	Description of experimental design			
2013	Preliminary experiment					
	Before maize sowing	Tillage	Rotary tillage	Rotary tillage	Rotary tillage	
		Mulching	New plastic mulching	New plastic mulching	New plastic mulching	
	After maize harvest	Tillage	No tillage	No tillage	Conventional tillage	
		Mulching	Residual plastic mulching	Residual plastic mulching	Plastic was removed	
2014	Real experiment					
		Treatment code	NM	RM	CM	
	Before maize sowing	Tillage	No tillage	Rotary tillage	Rotary tillage	
		Mulching	Residual plastic mulching	Old plastic was removed and new plastic was mulched		New plastic was mulched
	After maize harvest	Tillage	No tillage	No tillage	Conventional tillage	
		Mulching	Residual plastic mulching	Residual plastic mulching		Plastic was removed
2015	Real experiment					
		Treatment code	RM		NM	CM
	Before maize sowing	Tillage	Rotary tillage	No tillage	Rotary tillage	
		Mulching	Old plastic was removed and new plastic was mulched	Residual plastic mulching		New plastic was mulched
	After maize harvest	Tillage	No tillage	No tillage	Conventional tillage	
		Mulching	Residual plastic mulching	Residual plastic mulching	Plastic was removed	
2016	Real experiment					
		Treatment code	NM		RM	CM
	Before maize sowing	Tillage	No tillage	Rotary tillage	Rotary tillage	
		Mulching	Residual plastic mulching	Old plastic was removed and new plastic was mulched	New plastic was mulched	
	After maize harvest	Tillage	No tillage	No tillage	Conventional tillage	
		Mulching	Residual plastic mulching	Residual plastic mulching	Plastic was removed	

In this experiment, *Xian-yu* no. 335 maize (*Zea mays* L.) variety was applied in northwestern China (Hu et al., 2020). Maize was planted in mid-to-late April and harvested in mid-to-late September. Each plot size of this field experiment was 48 m^2^. The sowing ratio of maize was 8.25 plants m^–2^. Only nitrogen (urea) and phosphorus (diammonium phosphate) were applied in this experiment, because the area is rich in potassium; the application ratio of nitrogen (pure N) and phosphorus (P_2_O_5_) fertilizers for maize was 450 and 225 kg ha^–1^, across the three treatments. Across the maize growing season, 135, 270, and 45 kg ha^–1^ of pure N was applied at the sowing, jointing, and filling stages of maize, respectively; all P_2_O_5_ was applied at the maize sowing stage. Supplementary irrigation quota followed the level of local farmers; all plots received 900, 750, 900, 750, and 750 m^3^ ha^–1^ of irrigation were applied at the jointing, pre-heading, silking, flowering, and filling stages of maize.

### Experimental Data Acquisition

#### Air and Soil Temperature

Air temperature was automatically recorded by a small and simple weather station (BN-QX001, Boen, Beijing, China). Soil temperature was measured by geothermal meter (CJ-69, Licheng, Hengshui, China) on 7-day intervals in 5- to 25-cm soil layer with 5-cm intervals, at 08:00, 14:00, and 18:00 h in each of the measuring days, in maize growing season. Soil temperature in 5–25 cm at 08:00, 14:00, and 18:00 h was the average of each soil depth at every measuring day. Soil temperature in 5, 10, 15, 20, and 25 cm was the average of each measuring time at every measuring day. In addition, the difference value between air and soil temperatures was calculated in 5- to 25-cm soil layers in each growth period of different treatments.

#### Soil Accumulated Temperature

Soil accumulated temperature (SAT;°C) was calculated by the following equation:

$$SAT=\sum_{i=1}^{n}\left(ST_{i}\times D_{i}\right)$$

where *ST*_*i*_ (°C) is soil temperature at various growing stages of maize and *Di* (days) is the measuring interval time.

#### Soil Moisture

Soil gravimetric water content (GWC;%) was measured in 2-week intervals from maize sowing to harvesting with the oven-drying method throughout a sampling soil layer of 0–30 cm in a 10-cm increment. It was measured with a neutron probe (NMM503DR, Concord, CA, United States) throughout a sampling soil layer of 30–120 cm in a 30-cm increment. The oven-drying method was in line with the neutron probe measurements for the measuring places and times. Because a fully mulched system with plastic is used for maize cultivation in arid regions of northwestern China, all soil moisture samples were collected from the section of the plastic mulching.

#### Crop Water Consumption Characteristics

The WC modulus coefficient (WCMC;%) was calculated by WC of various maize growing stages divided by total WC across the entire maize growing season. The following equation is used to calculate WCMC (Cazcarro et al., 2013):

(1) $$WCMC_{i}=WC_{i}/WC_{t}\times 100\%$$

where *WC*_*i*_ (mm) is WC at various maize growing stages and *WC*_*t*_ (mm) is the total WC.

Meanwhile, the calculation equations of *WC*_*i*_ and *WC*_*t*_ are as follows (Lian et al., 2016):

(2) $$WC_{i}=P_{i}+I_{i}+\Delta SWS_{i}$$

(3) $$WC_{t}=\sum_{i}^{n}WC_{i}$$

where *P* and *I* are precipitation and irrigation, respectively, of each maize growing stage (mm); Δ*SWS* is the difference value of soil water storage (mm) in 0- to 120-cm soil layer between the pre- and post-growing stages of maize; and *i* represents the various maize growing stages.

#### Maize Grain Yield and Water Use Efficiency

When maize reached physiological maturity, three rows of unsampled maize plants were selected from each plot, and 3-m length was harvested from each row to evaluate the grain yield of maize. The harvested grain from each plot was weighed, and its moisture content was measured using a grain moisture meter (PM-8188, Shanghai, China). Grain yield was calculated at 13% moisture content, after threshing, cleaning, and air-drying. Use the following equation to calculate water use efficiency (WUE) (kg ha^–1^ mm^–1^):

$$\text{WUE}=\mathrm{Grain}\mathrm{yield}\left(\mathrm{kg}{\mathrm{ha}}^{-1}\right)/WC_{t}\left(\mathrm{mm}\right)$$

#### Economic Benefit

The major inputs included labor, machine, plastic, seeds, fertilizers, and irrigation and were recorded in each year. The major output included the economic value on grain and straw for maize (based on the local real-time prices at the time of the experiment). The net income (NI) was determined by calculating the difference between the values of total output and total input, in each treatment. The benefit dominance index (BDI) was determined by calculating the ratio of the net income per unit area of maize production in the experimental area to the average net income per unit area of maize in China; and the data come from the China Statistical Yearbook.

### Statistical Analysis

All data were analyzed using Statistical Analysis Software (SPSS 17.0, Inc., Chicago, IL, United States). Analysis of variance (ANOVA) was used to assess the significance of fixed effects, and means of treatments were compared using Fisher’s protected least significant difference (LSD). Year and treatment were considered fixed effects, and replication was considered a random effect. The year × treatment interaction and the main effects of year and treatment were assessed using ANOVA. The significances among treatments were presented at *P* < 0.05. Redundancy analysis (RDA) was conducted using the “lavaan” package (Rosseel, 2012) in R version 3.3.3 (R Foundation for Statistical Computing, Vienna, Austria, 2013). Correlation analysis based on Pearson’s correlation coefficients was performed between grain yield (GY), WUE, and net income (NI) and climatic and environmental factors.

## Results

### No Tillage With Plastic Re-Mulching Optimized Soil Temperature

#### Dynamics of Soil Temperature Across the Entire Maize Growing Season

**FIGURE 2:**
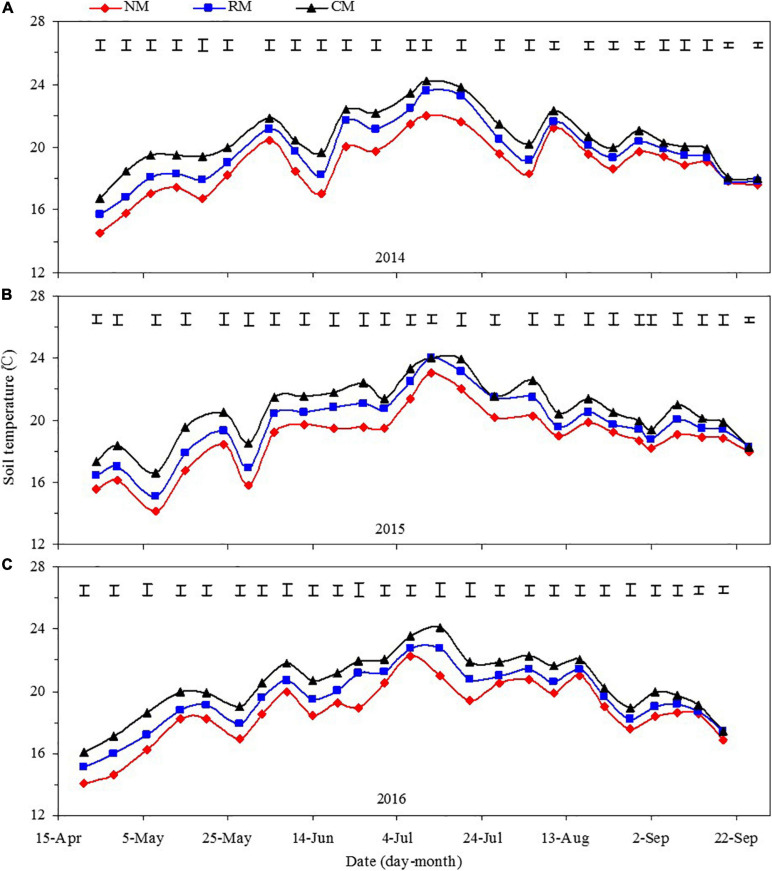
Dynamics of soil temperature in the 5–25 cm soil layer in maize field as affected by plastic mulching practices across the entire growing season in 2014 **(A)**, 2015 **(B)**, and 2016 **(C)**. NM, no tillage with plastic re-mulching; RM, no tillage with plastic mulching after maize was harvested in autumn and new plastic was mulched in next spring after the old plastic was removed off the field and rotary ploughed; CM, conventional deep tillage (the depth is 30 cm) after maize was harvested and the old plastic was removed off the field in autumn, and new plastic was mulched in next spring before maize sowing. The length of vertical bars represents the magnitude of the least significant difference (LSD) at *P* = 0.05 among treatments within a measurement date. NS, no significant difference at the 0.05 probability levels.

Plastic mulched management practices had a significant effect on soil temperature across the 5- to 25-cm soil layer; but year and year × treatment interaction had no significant effect on it (Figure 2). From maize sowing to big-flare stage, mean soil temperature in the 5- to 25-cm soil profile was decreased by 1.98–2.43°C and 0.91–1.36°C with NM and RM, compared with CM, respectively; and NM decreased soil temperature by 0.90–1.20°C over RM. From maize big-flare to silking stage, mean soil temperature in the 5- to 25-cm soil profile was decreased by 1.70–2.30°C and 1.10–1.40°C with NM as compared with CM and RM, respectively. With the advancement of maize growing period, the influence of plastic management on soil temperature decreased gradually. Across the maize reproductive period, NM decreased mean soil temperature in the 5- to 25-cm soil profile by 0.91–1.63°C as compared with CM, but no significant difference was found between CM and RM. Although the soil temperature of NM during the period from sowing to jointing was lower than that of RM and CM, which delayed the emergence of maize seedlings, the soil temperature of NM during the period from silking to filling was lower, which prevented senescence of roots or leaves for maize, and maintained normal grain filling.

#### Soil Temperature at Each Time of the Measuring Day

**FIGURE 3:**
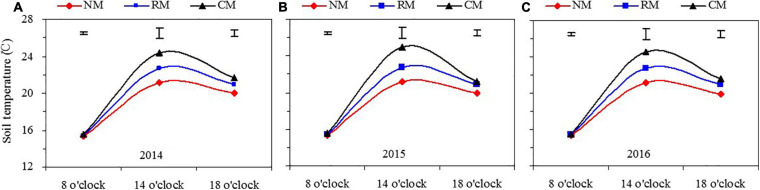
Dynamics of daily soil temperature in the 5–25 cm soil layer in maize field as affected by plastic mulching practices across the entire growing season in 2014 **(A)**, 2015 **(B)**, and 2016 **(C)**. NM, no tillage with plastic re-mulching; RM, no tillage with plastic mulching after maize was harvested in autumn and new plastic was mulched in next spring after the old plastic was removed off the field and rotary ploughed; CM, conventional deep tillage (the depth is 30 cm) after maize was harvested and the old plastic was removed off the field in autumn, and new plastic was mulched in next spring before maize sowing. The length of vertical bars represents the magnitude of the least significant difference (LSD) at *P* = 0.05 among treatments within a measurement time. NS, no significant difference at the 0.05 probability levels.

Soil temperature at the 5- to 25-cm soil layer was measured for the three plastic mulching treatments; and year and year × treatment interaction had no significant on soil temperature (Figure 3). At 08:00 h, plastic mulched management practices had no significant effect on mean soil temperature in 2014–2016. At 14:00 h, soil temperature of NM and RM was 3.15–3.38°C and 1.58–1.83°C lower than that of CM, respectively, and soil temperature with NM was 1.54–1.58°C less than that of RM. Similar to 14:00 h, at 18:00 h, soil temperature of NM and RM was 1.63–1.87°C and 0.66–0.87°C less than that of CM, respectively, and soil temperature with NM was 0.96–1.00°C less than that of RM. The results indicated that NM had the effects on preserving soil heat at low-temperature stage and reducing soil heat at the high-temperature stage in a day.

#### Vertical Variation of Soil Temperature Across the Soil Profile

**FIGURE 4:**
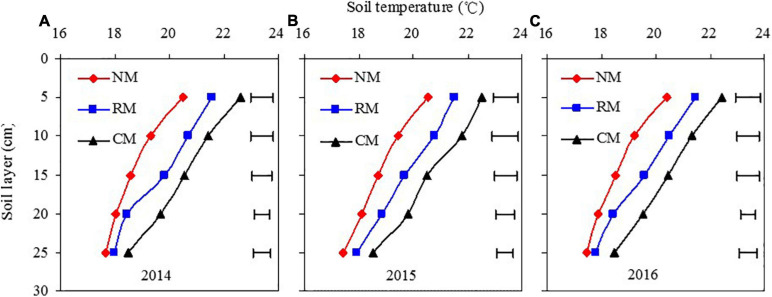
Soil temperature by soil layer in maize field as affected by plastic mulching practices across the entire growing season soil temperature by soil layer in maize field as affected by plastic mulching practices across the entire growing season in 2014 **(A)**, 2015 **(B)**, and 2016 **(C)**. NM, no tillage with plastic re-mulching; RM, no tillage with plastic mulching after maize was harvested in autumn and new plastic was mulched in next spring after the old plastic was removed off the field and rotary ploughed; CM, conventional deep tillage (the depth is 30 cm) after maize was harvested and the old plastic was removed off the field in autumn, and new plastic was mulched in next spring before maize sowing. The length of horizontal bars represents the magnitude of the least significant difference (LSD) at *P* = 0.05 among treatments within a soil layer.

There was no significant year and year × treatment interaction in affecting soil temperature across the 5- to 25-cm soil profile. All treatments followed a similar trend; soil temperature of NM treatment was significantly lower than that with CM, at a measured soil layer (Figure 4). At the 5-cm soil layer, soil temperature of NM and RM treatments was 1.96–2.10°C and 0.97–1.04°C lower than that of CM, respectively, and was 0.99–1.06°C of NM lower than that of RM, in 2014–2016. Similarly, NM and RM treatments reduced soil temperature by 2.11–2.36°C and 0.73–1.01°C as compared with CM, respectively, and NM reduced by 1.27–1.39°C in comparison with RM, at the 10-cm soil layer. At the 15-cm soil layer, NM and RM treatments decreased soil temperature by 1.77–1.95°C and 0.73–0.86°C over CM, respectively, and NM reduced by 0.96–1.08°C over RM. However, the difference on soil temperature among three treatments gradually declined at deeper soil layers at 20 and 25 cm, and NM decreased soil temperature by 1.63–1.71°C and 0.83–1.09°C at 20- and 25-cm soil layers, respectively, as compared with CM; but no significant differences were found between NM and RM. The results showed that the influence of plastic mulch management practices on soil temperature of maize farmland mainly occurred in 5- to 15-cm soil layer.

#### The Difference Between Air and Soil Temperature in the 5- to 25-cm Soil Layer

The influence of various plastic mulching management practices on soil temperature stability of maize field can be estimated by the difference between air and soil temperature in a soil layer of 5–25 cm (Table 2). From maize sowing to jointing stage, soil temperature in the 5- to 25-cm soil layer of CM treatment was greater than that of air temperature in the 3 years, and NM and RM treatments had greater soil temperature than air temperature in 2014 and 2016; and there were inter-annual differences between NM, but the absolute values of air–soil temperature difference with NM were smaller than those with RM and CM in 2014 and 2016, showing that the change of soil temperature was relatively stable with NM. From maize jointing to early-filling stage, soil temperature in the 5- to 25-cm soil layer of three treatments was lower than air temperature in the 3 years, and the difference between air and soil temperature was increased. This was because no tillage had the effect of decreasing soil temperature. Most importantly, during maize filling stage, the absolute value of air–soil temperature difference with NM was smaller than that with RM and CM in 2014–2016, showing that the change of soil temperature was relatively stable with NM. Compared with RM and CM treatments, the NM treatment had the effect of preserving soil heat across the low-temperature season and being relatively cool across the high-temperature season. It is an important regulatory mechanism to reduce the excessive influence of air temperature sudden change on the maize growth and development.

**TABLE 2:** The difference value (°C) between air temperature and soil temperature in the 5- to 25-cm soil layer as affected by plastic management across the maize growth stages from 2014 to 2016 in an arid oasis region.

Year	Treatment^a^	Maize growth stages^b^				
		Sowing—jointing	Jointing—big flare	Big flare—silking	Silking—early filling	Early filling—full ripe
2014	*NM*	−2.04^c^	2.12	1.70	3.15	–0.92
	*RM*	–3.11	1.02	0.30	2.42	–1.35
	*CM*	–4.48	0.11	–0.50	1.52	–1.83
2015	*NM*	1.14	1.49	0.96	3.00	–0.55
	*RM*	0.21	0.30	–0.14	2.01	–1.12
	*CM*	–1.11	–0.90	–0.74	1.36	–1.74
2016	*NM*	–0.24	1.15	2.83	2.74	–0.29
	*RM*	–1.17	0.25	1.52	2.12	–0.81
	*CM*	–2.30	–0.83	0.53	1.21	–1.34

*^*a*^NM, no tillage with plastic re-mulching; RM, no tillage with plastic mulching after maize was harvested in autumn and new plastic was mulched in next spring after the old plastic was removed off the field and rotary ploughed; CM, conventional deep tillage (the depth is 30 cm) after maize was harvested and the old plastic was removed off the field in autumn, and new plastic was mulched in next spring before maize sowing. ^*b*^The sampling dates were April 22, May 27, June 19, July 23, August 6, and October 1, 2014; April 23, May 30, June 21, July 27, August 9, and September 30, 2015; and April 19, May 28, June 26, July 20, August 8, and September 20, 2016. ^*c*^Soil temperature stability was judged by the absolute values between air and surface soil temperature; therefore, it is not suitable for statistical analysis.*

#### Soil Accumulated Temperature Was Affected by Plastic Mulched Management

Plastic mulched management had a significant difference on SAT during the entire growing season and each growing stage of maize, but there was no significant year in affecting SAT in each growing stage of maize; and year × treatment interaction in affecting SAT from the maize sowing to big-flare stage is significant, but other growing stages did not reach significance (Table 3). From the maize sowing to big-flare stage, the SAT of NM and RM treatments was decreased by 9.6–13.0% and 4.4–7.3% as compared with the CM control, and NM reduced SAT by 4.6–6.2% as compared with the RM. From maize big-flare to early-filling stage, only NM decreased SAT by 7.0–10.1% over CM, but no significant difference was found between RM and CM. However, across the filling stage, only NM decreased SAT by 5.9% in 2015 as compared with CM, but no significance in 2014 and 2016. In terms of entire maize growing season, the SAT value of NM was decreased by 8.4–8.8% as compared with CM.

**TABLE 3:** Soil accumulated temperature (°C) of each growth stage in maize with different plastic management practices from 2014 to 2016 in an arid oasis region.

Year	Treatment^a^	Maize growth stages^b^					Whole growth period
		Sowing—jointing	Jointing-big flare	Big flare—silking	Silking—early filling	Early filling—full ripe	
2014	NM	472c^c^	641b	573b	433b	904a	3,023b
	RM	503b	678ab	610a	449a	925a	3,166ab
	CM	543a	709a	632a	469a	948a	3,301a
2015	NM	535c	619c	516b	417b	849b	2,936b
	RM	566b	659b	542ab	438ab	875ab	3,079ab
	CM	610a	698a	557a	451a	902a	3,218a
2016	NM	553b	578b	633b	428b	742a	2,934b
	RM	585ab	606ab	674b	441ab	763a	3,068ab
	CM	623a	639a	704a	460a	785a	3,211a
*P*-value^d^							
Year (Y)		NS	NS	NS	NS	NS	NS
Treatment (T)		***	***	**	**	*	**
Y × T		*	*	NS	NS	NS	NS

^*a*^NM, no tillage with plastic re-mulching; RM, no tillage with plastic mulching after maize was harvested in autumn and new plastic was mulched in next spring after the old plastic was removed off the field and rotary ploughed; CM, conventional deep tillage (the depth is 30 cm) after maize was harvested and the old plastic was removed off the field in autumn, and new plastic was mulched in next spring before maize sowing. ^*b*^The sampling dates were April 22, May 27, June 19, July 23, August 6, and October 1, 2014; April 23, May 30, June 21, July 27, August 9, and September 30, 2015; and April 19, May 28, June 26, July 20, August 8, and September 20, 2016. ^*c*^Within a column for a given year, means followed by different lowercase letters are significantly different at *P* < 0.05. ^d^*, **, and *** Significant differences at the 0.05, 0.01, and 0.001 probability levels, respectively. NS, no significant differences at the 0.05 probability levels.

In other words, SAT with the NM treatment was decreased until maize early-filling stage, but there was almost no difference after maize early-filling stage. Although NM reduced the total SAT across the entire maize growing season, it had little influence on the SAT during the filling stage of maize and weakened the influence of SAT on the yield formation of maize.

### No Tillage With Plastic Re-Mulching Regulated Soil Moisture Status

#### Dynamics of Soil Water Content Across the Entire Maize Growing Season

**FIGURE 5:**
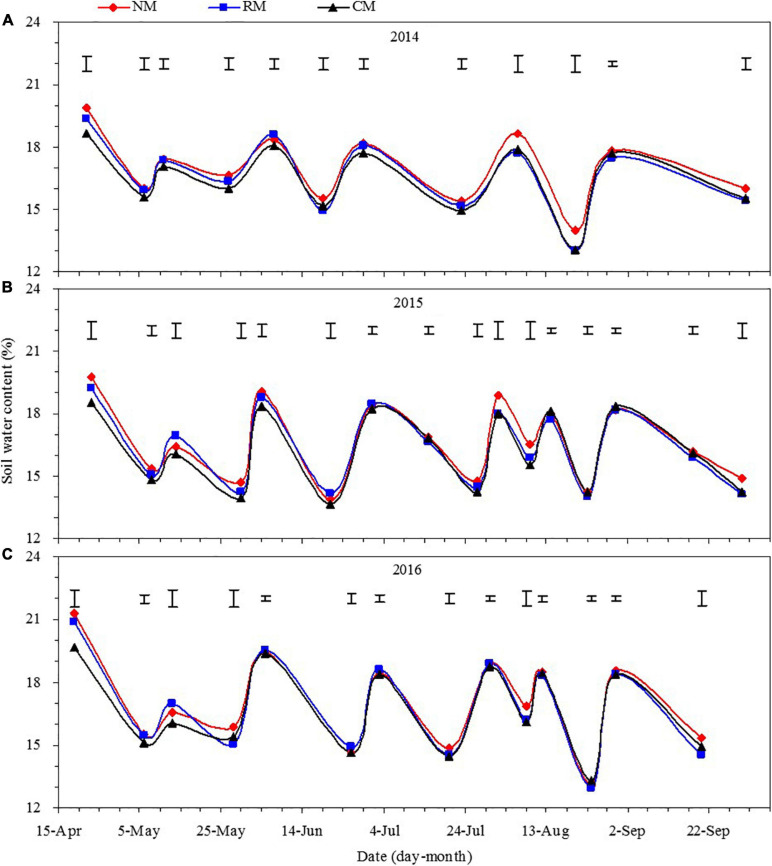
Dynamics of soil water content in the 0–120 cm soil layer in maize field as affected by plastic mulching practices across the entire growing season in 2014 **(A)**, 2015 **(B)**, and 2016 **(C)**. NM, no tillage with plastic re-mulching; RM, no tillage with plastic mulching after maize was harvested in autumn and new plastic was mulched in next spring after the old plastic was removed off the field and rotary ploughed; CM, conventional deep tillage (the depth is 30 cm) after maize was harvested and the old plastic was removed off the field in autumn, and new plastic was mulched in next spring before maize sowing. The length of vertical bars represents the magnitude of the least significant difference (LSD) at *P* = 0.05 among treatments within a measurement date. NS, no significant difference at the 0.05 probability levels.

The soil water content in the 0- to 120-cm soil layer during the entire maize growing season was mainly affected by irrigation, and the soil water content after irrigation was significantly greater than that before irrigation (Figure 5). In addition, soil water content in each of maize growing stage was affected by plastic mulched management practices, but the year × treatment interaction was not significant. From maize sowing to seedling stage, mean soil water content across the 0- to 120-cm soil layer was increased by 6.6–8.4% and 4.9–6.0% with NM and RM, compared with CM, respectively (Figure 5). However, plastic mulched management had no significant effect on it across maize jointing to silking stage. From maize silking to early-filling stage, NM treatment had greater soil water content by 4.8–5.6% than CM. After maize early-filling stage, soil water content of maize field had not been affected by plastic mulched management practices. The above results show that NM favorably stored more soil water from maize silking to early-filling stage, which compensated for the water requirement for the vigorous growth of maize plants across the filling stage.

#### Vertical Variation of Soil Water Content Across the Soil Profile

**FIGURE 6:**
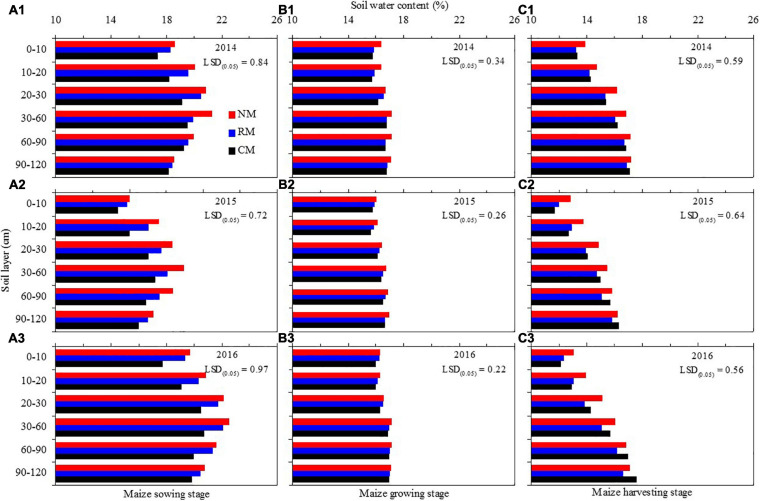
Soil water content by soil layer in maize field as affected by plastic mulching practices during different growth periods in 2014–2016. Soil water content before maize sowing **(A1–A3)**, maize growth period **(B1–B3)**, and after maize harvesting **(C1–C3)**. NM, no tillage with plastic re-mulching; RM, no tillage with plastic mulching after maize was harvested in autumn and new plastic was mulched in next spring after the old plastic was removed off the field and rotary ploughed; CM, conventional deep tillage (the depth is 30 cm) after maize was harvested and the old plastic was removed off the field in autumn, and new plastic was mulched in next spring before maize sowing. The value in each figure is the least significant difference (LSD) of all treatments among soil depths at *P* < 0.05.

Across the 0- to 120-cm soil profile, soil water contents of maize field increased with soil layer for all the treatments before maize sowing (Figures 6A1–A3). Compared with CM treatment, NM and RM increased soil water content in the 0- to 30-cm soil layer by 7.2–11.2% and 5.2–9.2%, respectively, in the 3 years. Similarly, soil water content of NM was increased by 6.7–9.1% in 30–90-cm soil layer in comparison with CM, but no significant difference was found between RM and CM. When below 90 cm, the effect of plastic mulched management on soil water content in maize field was not significant.

Soil water content of maize field increased with soil layer for all the treatments across the 0- to 120-cm soil layer during maize growing season (Figures 6B1–B3). However, plastic mulched management had no significant effect on the soil water content of maize field at a given whole soil layer.

In terms of maize harvest, soil water content of NM was increased by 4.5–10.2% in 0–60-cm soil layer as compared with CM control, but there was no significant difference found between RM and CM (Figures 6C1–C3). When below 60-cm soil layer, there was no significant difference on soil water content of maize field among the three plastic mulched treatments.

#### Plastic Mulched Management Optimized Crop Water Consumption Characteristics

Although plastic mulched management had no significant difference on WC during the entire growing season of maize, it had a significant influence on the WC and WCMC in each maize growing stage, but the year × treatment interaction was not significant for WC and WCMC in each maize growing stage (Table 4). From the maize sowing to jointing stage, the WC of NM and RM treatments was 9.5–20.0% and 6.7–14.2% greater than that of CM treatment; also, WCMC was greater by 7.1–24.7% and 5.7–15.7%, respectively, and no significant differences were found between NM and RM. From the maize jointing to big-flare stage, in 2015 and 2016, NM and RM treatments increased WC by 5.3–8.8% and 6.6–12.6% and increased WCMC by 6.5–9.5% and 5.6–14.1%, respectively, compared with CM; and NM had a tendency to reduce WC and reduced by 6.5% in 2016. NM treatment reduced WC and WCMC by 6.9–10.9% and 4.1–12.7% in comparison with CM, but there was no significant difference found between RM and CM, across the stage of big flare to early filling. However, from the maize early-filling to full-ripe stage, the NM treatment had a greater WC by 5.7–13.6% and 4.9–5.4% than RM and CM, respectively, and NM increased WCMC by 4.3–16.5% compared with RM and increased WCMC by 9.5% compared with CM only in 2016.

**TABLE 4:** Water consumption and water consumption modulus coefficient of maize at each of growth stage with different plastic management practices from 2014 to 2016 in an arid oasis region.

Year	Treatment^a^	Maize growth stages^b^										Whole growth period
		Sowing—jointing		Jointing—big flare		Big flare—silking		Silking—early filling		Early filling—harvesting		
		WC1 (mm)	WCMC1 (%)	WC2 (mm)	WCMC2 (%)	WC3 (mm)	WCMC3 (%)	WC4 (mm)	WCMC4 (%)	WC5 (mm)	WCMC5 (%)	ET (mm)
2014	NM	72a^c^	10.1a	129ab	18.2ab	146a	20.6a	70b	9.9c	292a	41.1a	709a
	RM	68a	9.7a	132a	18.9a	150a	21.5a	73ab	10.5b	276b	39.5b	700a
	CM	61b	8.8b	125b	18.0b	148a	21.3a	79a	11.3a	283ab	40.7ab	696a
2015	NM	107a	16.6a	145a	22.3a	93b	14.4b	72b	11.2c	230a	35.5a	647a
	RM	105a	16.4a	142a	22.1a	101a	15.8a	77ab	12.0b	215b	33.7b	639a
	CM	98b	15.5b	133b	21.0b	102a	16.1a	81a	12.8a	219b	34.6ab	634a
2016	NM	124a	18.7a	143b	21.6a	105b	15.8b	76b	11.6a	239a	36.1a	660a
	RM	118a	17.4b	153a	22.6a	116a	17.2a	80ab	11.9a	210c	31.0c	677a
	CM	103b	15.0c	136c	19.8b	112a	16.4ab	83a	12.1a	227b	33.0b	686a
*P*-value^d^												
Year (Y)		NS	NS	NS	NS	NS	NS	NS	NS	NS	NS	NS
Treatment (T)		*	*	**	**	*	*	**	**	**	**	**
Y × T		NS	NS	NS	NS	NS	NS	NS	NS	NS	NS	NS

^*a*^NM, no tillage with plastic re-mulching; RM, no tillage with plastic mulching after maize was harvested in autumn and new plastic was mulched in next spring after the old plastic was removed off the field and rotary ploughed; CM, conventional deep tillage (the depth is 30 cm) after maize was harvested and the old plastic was removed off the field in autumn, and new plastic was mulched in next spring before maize sowing. ^*b*^The sampling dates were April 22, May 27, June 19, July 23, August 6, and October 1, 2014; April 23, May 30, June 21, July 27, August 9, and September 30, 2015; and April 19, May 28, June 26, July 20, August 8, and September 20, 2016. ^*c*^Within a column for a given year, means followed by different lowercase letters are significantly different at *P* < 0.05. ^d^* and ** Significant differences at the 0.05 and 0.01 probability levels, respectively. NS, no significant differences at the 0.05 probability levels.

When compared with the CM treatment, WC with the NM treatment was increased until maize big-flare stage, decreased from maize big-flare to early-filling stage, and increased after the maize early-filling stage. This effectively coordinated water demand contradiction of maize at early and late stages and created a more optimal water balance for maize growth.

### No Tillage With Plastic Re-Mulching Maintained High Grain Yield and Water Use Efficiency, and Increased Economic Benefits

**FIGURE 7:**
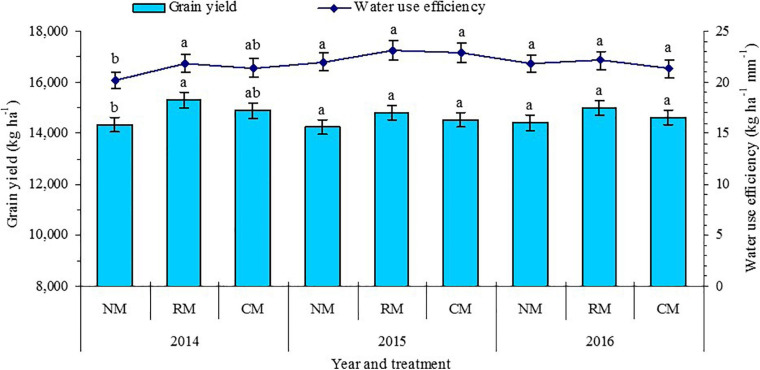
Grain yield and water use efficiency of maize as affected by plastic mulching practices in 2014–2016. NM, no tillage with plastic re-mulching; RM, no tillage with plastic mulching after maize was harvested in autumn and new plastic was mulched in next spring after the old plastic was removed off the field and rotary ploughed; CM, conventional deep tillage (the depth is 30 cm) after maize was harvested and the old plastic was removed off the field in autumn, and new plastic was mulched in next spring before maize sowing. Within a year for a given figure pane, different lowercase letters indicate treatment means that are significantly different at *P* < 0.05. Error bars denote standard errors of the means.

The effects of plastic mulched management on grain yield of maize varied from 2014 to 2016 (Figure 7). Only in 2014, NM treatment had lower grain yield and WUE of maize by 6.9% and 5.6% than treatment but had no significant difference between NM and CM treatments. In 2015 and 2016, plastic mulched management had no significant difference on grain yield and WUE of maize. The results showed that the higher grain yield and WUE of maize could be maintained by no tillage with plastic re-mulching, and it was a feasible measure to reduce the input of plastic for maize cultivation in the oasis irrigated area where maize cultivation must depend on plastic mulching.

Compared with CM control, NM treatment mainly decreased the input costs for plastic and machine operations (Table 5). The total input costs for the NM treatment were 23.9–27.6% (317–431 USD ha^–1^) lower than those of RM treatment and were 26.2–28.2% (417–443 USD ha^–1^) lower than those of CM treatment. Plastic mulched management practices had no significant effect on total output value. By contrast, due to the decreased total input costs with NM, the net income with NM increased by 6.4–11.0% (228–346 USD ha^–1^) compared with CM in the 3 years, and the net income with NM increased by 6.6–7.7% (209–250 USD ha^–1^) compared with RM in 2015 and 2016. Among all the plastic mulched management practices, the output/input ratio with NM was 22.9–32.9% and 30.5–36.3% higher than that with RM and CM treatments, respectively. The fact that the NM treatment produced a higher net income in comparison with RM and CM treatments could be attributed to its lower total input costs.

**TABLE 5:** Analysis on economic benefit for maize production under different plastic management practices from 2014 to 2016 in an arid oasis region.

Year	Treatment^a^	Input value (USD ha^–1^)				Output value (USD ha^–1^)			NI (USD ha^–1^)	Benefit over CM	TO/TI	RR (%)	BDA
		*P*^b^	LM	SFI	Total	Grain	Straw	Total					
2014	NM	0	244	939	1,183b	4,662b	328b	4,990b	3,807a	228	4.22a	322a	24.3a
	RM	159	476	919	1,554a	4,983a	349a	5,333a	3,779a	199	3.43b	243b	24.1a
	CM	159	525	919	1,603a	4,847ab	335ab	5,182ab	3,579b	0	3.23c	223c	22.8b
2015	NM	0	236	894	1,130b	4,297a	321a	4,618a	3,488a	346	4.09a	309a	23.0a
	RM	153	531	877	1,561a	4,468a	331a	4,799a	3,238b	96	3.07b	207b	21.4b
	CM	153	542	877	1,573a	4,382a	334a	4,715a	3,142b	0	3.00b	200b	20.8b
2016	NM	0	223	858	1,081b	4,025a	434a	4,459a	3,378a	334	4.13a	313a	23.6a
	RM	145	501	840	1,487a	4,190a	466a	4,655a	3,169b	124	3.13b	213b	22.1b
	CM	145	513	840	1,498a	4,083a	459a	4,542a	3,044b	0	3.03b	203b	21.3b
*P*-value^c^													
Year (Y)					NS	NS	NS	NS	NS		*	*	NS
Treatment (T)					*	*	*	*	*		**	**	**
Y × T					NS	NS	NS	NS	NS		*	*	NS

^*a*^NM, no tillage with plastic re-mulching; RM, no tillage with plastic mulching after maize was harvested in autumn and new plastic was mulched in next spring after the old plastic was removed off the field and rotary ploughed; CM, conventional deep tillage (the depth is 30 cm) after maize was harvested and the old plastic was removed off the field in autumn, and new plastic was mulched in next spring before maize sowing. ^*b*^P, plastic; LM, labor and machine; SFI, seed, fertilizer, and irrigation; NI, net income; TO/TI, total output/total input; RR, rate of return; BDA, benefit dominance analysis. In 2014–2016, the labor input was calculated based on the local wages of 100 ¥ per farmer per 8 h; the price of irrigation was 0.3 ¥ m^–3^; the prices of plastic, diammonium phosphate, and urea were 13, 3.5, and 1.65 ¥ kg^–1^, respectively; the price of maize seeds was 28 ¥ kg^–1^. The cost of maize harvested and transported by machine was 1,500 ¥ ha^–1^. The prices of grain and straw for maize was, respectively, 2.00 ¥ kg^–1^ and 0.15 ¥ kg^–1^ in 2014; 1.92 ¥ kg^–1^ and 0.15 ¥ kg^–1^ in 2015; and 1.88 ¥ kg^–1^ and 0.20 ¥ kg^–1^ in 2016. The price expressed in USD was based on the exchange rate of 6.144 ¥ for every USD in 2014, 6.361 ¥ for every USD in 2015, and 6.731 ¥ for every USD in 2016. ^*c*^Within a column for a given year, means followed by different lowercase letters are significantly different at *P* < 0.05. ^d^* and ** Significant differences at the 0.05 and 0.01 probability levels, respectively. NS, no significant differences at the 0.05 probability levels.

Compared with CM control, the rate of return for maize production with NM treatment was increased by 44.1–54.5%, and it was increased by 32.4–48.8% compared with RM (Table 5). Similar to the rate of return, NM treatment had greater BDI by 6.4–11.0% than CM treatment. These results indicated that the maize production level of oasis irrigated area in northwest China was better than the national average level, and the benefit capacity for maize production under the NM treatment was the best.

### Relationships Between Grain Yield, Water Use Efficiency, Net Income, and Soil Hydrothermal and Climate Factors

**FIGURE 8:**
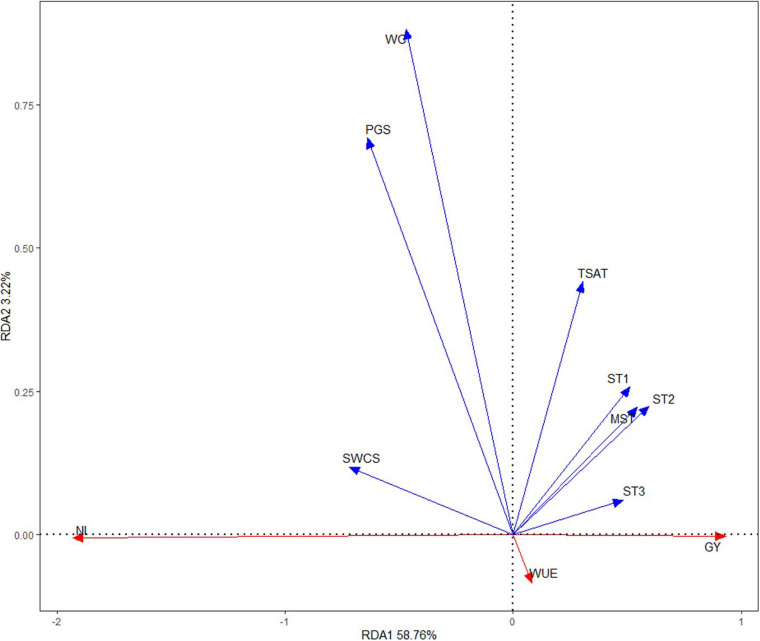
Redundancy analysis (RDA) based on the grain yield (GY), water use efficiency (WUE), net income (NI), and climate and soil hydrothermal factors at the Wuwei Experimental Station in northwestern China. SWCS, soil water content at sowing; WC, water consumption; ST1, soil temperature from seedling to jointing; ST2, soil temperature from big flare to early filling; ST3, soil temperature from early filling to full ripe; MST, mean soil temperature; TSAT, total soil accumulated temperature; PGS, precipitation across the maize growth season; AT, air temperature.

RDA was performed to represent the relationships between the grain yield (GY), WUE, net income (NI), and soil hydrothermal and climate factors (Figure 8). The results showed that the GY and WUE were positively correlated; but the GY and NI, and WUE and NI were negatively correlated. Overall, the soil hydrothermal and climate factors accounted for 58.76% of the variations in the GY, WUE, and NI. WC and precipitation across the maize growth season (PGS) had the strongest correlation (long arrows) with the GY, WUE, and NI, followed by soil water content at sowing (SWCS) and total SAT (TSAT) and then followed by soil temperature parameters (ST1, ST2, ST3, and MST).

Correlation analysis showed that all of the soil hydrothermal and climate factors were not significantly associated with the GY (Table 6). There was a significant negative correlation between WUE and WC, and precipitation across the maize growth season (PGS) but a significant positive correlation between WUE and air temperature (AT). NI was significantly positively correlated with soil water content at sowing (SWCS), WC, and PGS but negatively correlated with AT (*P* < 0.05). The results showed that no tillage with plastic re-mulching reduced the adverse effects on maize production by regulating the hydrothermal effect of soil.

**TABLE 6:** Correlation analysis between the grain yield, water use efficiency, net income, and soil hydrothermal and climate factors.

Indexes^a^	Soil hydrothermal factors^b^					Climate factors^c^			
	SWCS	WC	ST1	ST2	ST3	MST	TSAT	PGS	AT
GY	0.271^d^	0.334	0.418	0.454	0.318	0.424	0.525	0.383	–0.381
WUE	–0.308	−0.836**	0.256	0.346	0.369	0.308	0.050	−0.703*	0.699*
NI	0.806**	0.593*	–0.384	–0.462	–0.389	–0.419	–0.140	0.791**	−0.797**

^*a*^GY, grain yield; WUE, water use efficiency; NI, net income. ^*b*^SWCS, soil water content at sowing; WC, water consumption; ST1, soil temperature from seedling to jointing; ST2, soil temperature from big flare to early filling; ST3, soil temperature from early filling to full ripe; MST, mean soil temperature; TSAT, total soil accumulated temperature. ^*c*^PGS, precipitation across the maize growth season; AT, air temperature. ^*d*^The values are Pearson’s correlation coefficients. *P < 0.05 and **P < 0.001 indicate significant relationships.

## Discussion

### Plastic Mulched Management Optimized Soil Temperature

Temperature is one of the main factors on growth and development of crop (Porter and Gawith, 1999). Soil temperature is the basis for influencing the temperature variation received by crops, is a significant factor for holding crop root activity, and is of great importance to crop productivity in farmland (Stone et al., 1999). Various covering materials and covering patterns had different effects on soil temperature across some scientific findings (Chen et al., 2010; Li et al., 2013). Also, the same covering material has different thermal effects during various using ways in this study. This result is consistent with the previous findings, which found that soil temperature with no tillage was lower than conventional tillage (Drury et al., 1999; Subrahmaniyan and Zhou, 2008), and in the present research, no tillage with residual plastic re-mulching (NM treatment) reduced soil temperature as compared with CM treatment (conventional deep tillage after maize was harvested and the old plastic was removed off the field in autumn, and new plastic was mulched in next spring before maize sowing). This was likely because NM practice had more soil covered and the plastic prevents the exchange of water between the soil and the atmosphere; therefore, the latent heat flux and heat exchange between the soil and the atmosphere are decreased (Zhao et al., 2017). Also, these are because NM practice can intercept solar energy and prevent heat loss as re-radiation from the soil surface to the surrounding atmosphere (Ghawi and Battikhi, 2010). These findings have been validated in this research, as CM produced the highest soil temperature while NM produced the lowest soil temperature detected in 3 years.

Some findings confirmed that soils uncultivated in cold weather are warmer than soils cultivated in warm weather when compared with conventional deep tillage (Fabrizzi et al., 2005). Also, conventional tillage with annual new plastic mulch (CM) had a significant effect on increasing and decreasing rapidly soil temperature, with the increase or decrease of air temperature (Yin et al., 2016). This was because plastic covers the soils and blocks the passage of gas and water into the surrounding atmosphere, and the gap between the soil surface and the plastic can collect water and gas (Wu et al., 1996; Ghawi and Battikhi, 2010). However, for the no tillage with plastic re-mulching practice (NM), the integrity of plastic mulch was maintained at about 70%; thus, soil temperature of NM had a relatively small fluctuation with the change of air temperature, as compared with CM. In the present study, the results indicated that NM can optimize surface soil heat status of maize field, but the plastic mulched management practices had different effects on soil heat status across the measuring time of 1 day. Compared with CM, the range of soil temperature with NM treatment was increased by a small scope across the high-temperature season and decreased by a small scope across the low-temperature season. Therefore, NM enhanced the soil thermal preservation, and the stability of soil temperature changes. This may be because NM practice has a higher albedo and lower thermal conductivity than has CM, and soil temperature changes slowly with the change of solar radiation intensity (Wu et al., 1996); thus, the daily variation on soil temperature with NM was smaller than that with CM. Previous research confirmed that soil temperature in maize field was often greater than 40°C at noon with CM practice in July and August of each year (Yin et al., 2016, 2020a), which can reduce root activity, cause leaf rolling or senescence, and affect grain-filling, thus decreasing grain yield. On the contrary, NM practice had more effective regulating soil temperature of the maize field than CM practice, resulting in positive collaborative growth of maize with NM in this study. Moreover, for the NM practice, soil heat was maintained in the low-temperature season, and it was reduced across high-temperature season according to the value of difference between air and soil temperatures in this study. In particular, the absolute value of air–soil temperature difference with NM was smaller than that with other treatments, during maize filling stage, indicating that the change of soil temperature was relatively stable with NM for ensuring grain filling, which is an important regulatory mechanism on maize growth and development by decreasing the adverse influence resulting from the smaller temperature change.

### Plastic Mulched Management Regulated Soil Moisture

In recent years, plastic mulch is one of the main soil water conservation measures in the face of increasing shortage of water resources (Chai et al., 2014). It has been widely used in agricultural production and can enhance soil moisture retention and infiltration capacity (Zhou et al., 2009). Especially in the arid oasis irrigated regions of northwestern China, the warm-season maize cultivation relied on plastic mulching because the potential for soil evaporation is 20 times greater than precipitation per year (Chai et al., 2014). However, conventional maize production was covered with annual new plastic and deep tillage, which can lead to higher soil temperature of maize root zone from silking to filling stage, thus leading to root or leaf senescence and lowering maize production (Bu et al., 2013; Yin et al., 2020a). Therefore, plastic was reused in this study as a reduction input technique. In this study, three plastic mulched management practices had similar effects on conserving soil moisture across the maize growing season, and NM treatment stored more water than RM and CM from maize sowing to seedling stage and maintained for a longer time so as to be used in the vigorous growth of the later stage of maize. This was because the integrity of the plastic mulch was maintained at greater than 70% in NM treatment; in addition, a thin layer of soil covering the old plastic has the same inhibitory effect on soil evaporation as RM and CM treatments (Yin et al., 2018b).

In the present research, NM treatment can reduce soil temperature, slow growth of the maize, and consume less moisture and nutrients before big-flare stage. On the contrary, with the rise of air temperature, the remaining soil moisture and nutrients in the early growth stage boost the exuberant growth and development of maize. So WC with the NM treatment was increased until maize big-flare stage, decreased from maize big-flare to early-filling stage, and increased after the early-filling stage of maize, when compared with the CM treatment. This effectively coordinated water demand contradiction of maize at early and late stages and created a more optimal water balance for maize growth. The above results further concluded that NM was probably more resistant to drought stress because of the difference in soil moisture across the early- or late-growth stages, in comparison with CM. This was because soil water recharge relies on precipitation capture and irrigation conservation *via* previous residual plastic re-mulching in an arid environment (Zhao et al., 2017; Yin et al., 2020a). These further proved the possibility of using previous residual plastic re-mulching for maize cultivation in arid or semiarid areas.

### Grain Yield and Economic Benefit as Affected by Plastic Mulched Management

Many researchers have found that plastic mulching can boost maize productivity in arid oasis irrigated areas (Zhang et al., 2016; Zhao et al., 2017). However, annual mulched new plastic caused more and more serious ecological and environmental problems in farmland (Liu et al., 2014). For example, the maturity period of maize in Hexi Corridor of Gansu Province has been advanced from mid-October to early September and even late August, showing a trend of decreasing production, in the recent 10 years. In fact, this is premature senility rather than precocity. The main reasons are as follows: (1) extreme changes of high soil temperature and low temperature (Yin et al., 2016); (2) soil temperature in the exuberant growth period (flowering to filling stage) of maize reached above 40°C with annual mulched new plastic, which is significantly higher than the appropriate temperature threshold of 35°C for the normal growth and development of maize roots, and harmed maize root activity and caused premature senescence (Tao et al., 2013); (3) large amount of plastic input consumed the excessive soil water and nutrients, which caused soil microenvironment deterioration (Lee et al., 2018; Wang et al., 2020); and (4) annual mulched new plastic increased soil temperature, which led to the rapid growth of maize in the early stage and more consumption of nutrients and water, resulting in the phenomenon of lack of water and fertilizer in the late growth stage of maize (Bu et al., 2013). In this study, NM treatment can mitigate the above drawbacks, such as (1) achieve plastic reduction input and reduce the potential risk of overinvestment in plastic; (2) optimize the hydrothermal effect of soil and reduce the extreme high soil temperature, especially retained soil moisture during the fallow period, in this study; (3) effectively coordinated water demand contradiction of maize at early and late stages and created a more optimal water balance for maize growth, in this study; (4) this might be because NM treatment prolonged the maize growth period, delayed the functional period of green leaf, increased the leaf area index, and enhanced the accumulation and transformation of the photosynthetic product (Yin et al., 2020c); (5) no tillage with residual plastic film mulch may promote chlorophyll synthesis and increase photosynthesis of leaves *via* enhancing soil moisture conservation (Yang et al., 2018); and (6) this could partly be attributed to the increased transport of dry matter to the grain from the leaves, stems, and sheaths across the late maize growth stage. Meanwhile, in many studies on crop cultivation, the dry matter conversion was facilitated, and harvest index of crops was greater under no tillage treatments than conventional tillage, and this phenomenon was attributed to the suppression of early vegetative growth, while late reproductive growth is promoted with no tillage practice (Khan et al., 2014; Yin et al., 2017). Thus, NM treatment can still maintain high maize production as compared with CM, and it is feasible to apply no tillage with plastic re-mulching in Hexi oasis irrigation area.

Economic benefit is the ultimate goal of agricultural producers, and the improvement of the total output value is the basis of achieving higher net income. Plastics have played a very important role in addressing the problem of maize production safely and increasing farmers’ income in northwestern China (Xu et al., 2015; Wang et al., 2016). Moreover, no tillage can decrease operation times in the farmland, thus reducing the input costs of agricultural production for machinery, fuel, labor, and other equipment while enhancing agricultural productivity (Bueno et al., 2007; Yin et al., 2018a). The current research suggested that no tillage with plastic re-mulching (NM) decreased the total input cost values by 26.2–28.2% (417–443 USD ha^–1^) and increased the net income and the rate of return by 6.4–11.0% (228–346 USD ha^–1^) and 44.1–54.5%, thus increasing the output/input ratio by 30.5–36.3%, compared with conventional tillage with annual new plastic mulching (CM). Although resource’s inputs and the market prices for harvested grain tend to change over the years, there was no significant difference in total output value between NM and CM treatments, because they have no difference in yield. On the contrary, NM treatment can decrease the total input costs over CM, so NM treatment can improve the net income for maize production. NM treatment had greater BDI by 6.4–11.0% than CM treatment, which indicated that the maize production level of oasis irrigated area in northwest China was better than the national average level, and the benefit capacity for maize production under the NM treatment was the best. These results provide strong practical basis that the adaptation of no tillage with plastic re-mulching will maintain yield stability *via* optimizing the hydrothermal effect of soil while increasing the farmers’ income in oasis irrigated agricultural regions.

## Conclusion

No tillage with plastic re-mulching (NM treatment) was shown to be effective at regulating soil hydrothermal characteristics of maize production. The regulated effect allowed the growing of the maize to occur in a suitable moisture and heat conditions across its growing season. The NM treatment had the effect of retaining soil heat across the low-temperature season and reducing soil temperature across the high-temperature season, especially the absolute values of air–soil temperature difference with NM was smaller than those with other treatments, during maize filling stage, indicating that the change of soil temperature was relatively stable with NM for helping to ensure grain filling. It is an important regulatory mechanism on maize growth and development by decreasing the adverse influence resulting from the smaller temperature change. In addition, NM treatment increased WC before maize big-flare stage, decreased WC from big-flare to early-filling stage, and increased WC after early-filling stage, which effectively regulated the difference of water requirements of maize at different growing stages and created a more optimal water balance for maize growth. Thus, the NM treatment can still maintain the high yield and obtain higher net income and the rate of return for maize while reducing the input costs of plastic and machine operations. Collectively, the NM treatment, with its positive effect on regulating soil temperature and moisture to overcome heat stress and water shortage, can be utilized to maintain high maize productivity and reduce the input of plastic and machine operations in oasis irrigation regions where maize cultivation must depend on plastic mulching.

## Data Availability Statement

The original contributions presented in the study are included in the article.

## Author Contributions

QC and WY conceived and designed the experiment. WY, QC, and YG performed the statistical analyses. WY, YG, HF, FH, AY, CZ, and ZF were involved in field data collection. QC and JC critically reviewed the manuscript. All authors contributed to writing the manuscript and approved the final version of the manuscript.

## Conflict of Interest

The authors declare that the research was conducted in the absence of any commercial or financial relationships that could be construed as a potential conflict of interest.
